# Abnormal error processing in depressive states: a translational examination in humans and rats

**DOI:** 10.1038/tp.2015.54

**Published:** 2015-05-12

**Authors:** C Beard, R J Donahue, D G Dillon, A Van't Veer, C Webber, J Lee, E Barrick, K J Hsu, D Foti, F I Carroll, W A Carlezon Jr, T Björgvinsson, D A Pizzagalli

**Affiliations:** 1 Department of Psychiatry, Harvard Medical School/McLean Hospital, Belmont, MA, USA; 2 Research Triangle Institute, Research Triangle Park, NC, USA

## Abstract

Depression has been associated with poor performance following errors, but the clinical implications, response to treatment and neurobiological mechanisms of this post-error behavioral adjustment abnormality remain unclear. To fill this gap in knowledge, we tested depressed patients in a partial hospital setting before and after treatment (cognitive behavior therapy combined with medication) using a flanker task. To evaluate the translational relevance of this metric in rodents, we performed a secondary analysis on existing data from rats tested in the 5-choice serial reaction time task after treatment with corticotropin-releasing factor (CRF), a stress peptide that produces depressive-like signs in rodent models relevant to depression. In addition, to examine the effect of treatment on post-error behavior in rodents, we examined a second cohort of rodents treated with JDTic, a kappa-opioid receptor antagonist that produces antidepressant-like effects in laboratory animals. In depressed patients, baseline post-error accuracy was lower than post-correct accuracy, and, as expected, post-error accuracy improved with treatment. Moreover, baseline post-error accuracy predicted attentional control and rumination (but not depressive symptoms) after treatment. In rats, CRF significantly degraded post-error accuracy, but not post-correct accuracy, and this effect was attenuated by JDTic. Our findings demonstrate deficits in post-error accuracy in depressed patients, as well as a rodent model relevant to depression. These deficits respond to intervention in both species. Although post-error behavior predicted treatment-related changes in attentional control and rumination, a relationship to depressive symptoms remains to be demonstrated.

## Introduction

Cognitive impairment is a debilitating component of depression.^1, 2^ Deficits in cognitive control—the ability to modulate behavior in order to meet fluctuating environmental demands or internal goals—precede the onset of depressive symptoms,^3^ persist following symptom remission^4, 5^ and predict poor response to treatment.^6^ Cognitive control impairments may be particularly salient in the context of perceived failure.^7, 8, 9, 10, 11^ Indeed, several prior studies found that following an incorrect response—but not a correct response—performance (for example, accuracy) deteriorates in clinical and subclinical-depressed samples, a phenomenon described as ‘catastrophic response to perceived failure'.^8^ Despite these intriguing findings, compared with other cognitive control impairments, we know little about the clinical correlates and neurobiological substrates of abnormal post-error behavioral adjustments. To date, no studies have examined whether they can be ameliorated by treatment or whether they predict treatment response. Moreover, no studies have determined whether an analogous phenomenon exists in rodents, in part, because of difficulties in modeling depression in this species. The existence of these signs in rats in response to manipulations that produce depressive-like behaviors might facilitate translational studies and thus provide insight on mechanisms and sensitivity to existing or novel interventions.

The current study examined post-error behavioral adjustment in depression by integrating behavioral data from depressed patients undergoing treatment and from rodents (rats) given a manipulation known to produce hallmark signs of depression. First, in the human study, we examined the effect of treatment on post-error behavior elicited during an arrow flanker task. Treatment consisted of intensive cognitive-behavioral therapy (CBT) and pharmacological medication in a partial hospital setting. Although the evidence for a positive effect of depression treatment on cognitive impairments is mixed,^12, 13, 14^ we expected that patients would show improved post-error behavioral adjustments following treatment because CBT specifically targets negative cognitive schemas related to failure, and thus should decrease the impact of perceived failure on performance. Given that other types of cognitive impairment predict poor response to treatment, we also expected to find a positive relationship between pretreatment post-error behavioral adjustment and treatment outcomes. This was based on a two-part hypothesis: (1) post-error behavioral adjustments depend on the deployment of cognitive control and (2) participants who could capably exert cognitive control at baseline would be most likely to benefit from CBT.

We adopted a translational approach to investigate neurobiological mechanisms associated with abnormal post-error behavioral adjustments. To this end, we performed proof-of-concept secondary analyses of data from a rodent study^15^ to (1) examine whether post-error accuracy is impaired when rats are given a manipulation known to produce depressive-like behaviors, and (2) determine whether such effects could be mitigated by an intervention that produces antidepressant-like effects. Specifically, we examined the effects of corticotropin-releasing factor (CRF) on performance in the 5-choice serial reaction time task (5CSRTT)—a behavioral procedure widely used to quantify attention in rats.^16, 17^ CRF has a critical role in the physiological response to stress and has been implicated in the pathophysiology of anxiety and depression.^18, 19^ Indeed, individuals with mood disorders exhibit higher levels of CRF in cerebrospinal fluid.^20, 21^ CRF administration is known to induce depression- and anxiety-like phenotypes in rodents,^22, 23^ and we have previously found that CRF infusion degrades 5CSRTT performance, yielding decreases in correct responding, increased omission errors, increased latencies to respond correctly and increased time needed to complete the test session.^15^ Here, we present a novel analysis of CRF effects on post-error adjustments. We also examined the effect of a pharmacological intervention in the rats: specifically, we tested whether administration of JDTic—a kappa-opioid receptor (KOR) antagonist with antidepressant- and anxiolytic-like effects that can attenuate CRF-induced attention deficits^15, 24^—would likewise ameliorate post-error performance impairments.

## Study 1: Patients

### Patients and treatment

Data were collected from patients receiving treatment for a mood disorder at the Behavioral Health Partial Hospital Program at McLean Hospital, Belmont, MA, USA. The partial hospital delivers brief, intensive group, and individual CBT and pharmacological treatment to individuals with a range of psychiatric disorders (principally mood, anxiety, personality and psychotic disorders; see Beard and Björgvinsson^25^ for details). Individual treatment plans are constructed for each patient by clinical team managers who conduct initial intake assessments, develop an initial conceptualization, identify a problem list for patients and oversee all aspects of treatment in conjunction with a program psychiatrist. Treatment consists primarily of group CBT provided by psychologists, social workers, occupational therapists, nurses, postdoctoral and graduate level psychology trainees and mental health counselors. Patients attend up to five 50-min groups each day, 5 days per week (Monday–Friday), and 2–3 weekly individual CBT sessions. Finally, patients also meet with a program psychiatrist for medication management. The average duration of treatment is ~8 days. All patients received a similar type, intensity and duration of CBT (that is, primarily group format, daily and brief—<2 weeks) targeting depression.

Eligibility criteria included a current mood disorder and being stable enough to complete a research protocol (that is, not actively psychotic). Of 88 eligible participants, 63 had complete data, and the remaining 25 did not complete either the Time 1 or Time 2 flanker task for a variety of reasons (for example, scheduling conflicts). Of the participants with complete data, 31 did not meet quality control cutoffs for examination of post-error behavioral adjustments (see below and Supplementary Material), resulting in 32 final participants. Excluded participants did not differ from those in the final sample on baseline demographic or clinical variables, with the exception of lower scores on the BASIS-24 (Behavior and Symptom Identification Scale) relationship subscale, *t*(88)=2.31, *P*=0.023.

Participants were primarily single (*n*=23), female (*n*=22), middle-aged (*M*=29.97 years old, s.d.=12.00) and reported the following ethnoracial backgrounds: non-Latino white (*n*=25), multiracial (*n*=3), Latino (*n*=2) and no answer (*n*=2). Psychiatrists assigned diagnoses for all patients based on chart history and clinical interview. Mood disorders included major depressive disorder (*n*=21), bipolar I disorder (*n*=5), bipolar II disorder (*n*=2) and mood disorder not otherwise specified (*n*=4). All patients were depressed upon admission to the partial hospital. Of those participants with information on comorbid disorders from a structured interview (*n*=21), the most common current comorbid Diagnostic and Statistical Manual-IV Axis I diagnoses were generalized anxiety disorder (*n*=9), post-traumatic stress disorder (*n*=4), social anxiety disorder (*n*=4), panic disorder (*n*=4) and alcohol dependence (*n*=3). The average number of comorbid Axis I disorders was 1.75 (s.d.=1.77). Medication data were available from medical charts for 27 participants, and most of these individuals (*n*=23) were receiving pharmacological treatment upon admission to the partial hospital (antidepressant: *n*=19, antianxiety: *n*=11, mood stabilizer: *n*=9, antipsychotic: *n*=8, range=0–7 medications; M=2.44 and s.d.=1.63).

### Procedures

The local institutional review board approved all procedures, and patients provided informed written consent. Patients completed the flanker task on the following two occasions: on their first full day of treatment (that is, second day in the program; Time 1) and discharge day (Time 2). On average, these sessions were separated by 11.22 days (s.d.=2.92). Admission and discharge assessments included self-report measures collected and managed using REDCap electronic data capture tools hosted at McLean Hospital.^26^ Participants completed a structured diagnostic interview at Time 1. To improve the retention rate for the second experimental session, we began compensating participants near the end of data collection. Fourteen participants (seven with complete data and flanker data meeting quality control cutoffs) received $25 for completing the discharge flanker task (their findings did not differ from participants without compensation; findings available upon request).

### Instruments

Depressive symptoms were assessed with the Center for Epidemiologic Studies Depression Scale-10 (CESD-10).^27^ The revised BASIS-24 was used to assess depression, difficulty with relationships, self-harm, emotional lability, psychotic symptoms and substance abuse.^28^ Use of cognitive-behavioral skills was measured with the Cognitive Behavior Therapy Skills Questionnaire (CBTSQ-16).^29^ We assessed rumination with the Ruminative Responses Scale (RRS),^30^ which measures two types of rumination: *brooding*, which entails passively dwelling on the causes and consequences of low mood, and is considered a maladaptive form of rumination, and *reflection*, which is characterized by a more distanced, problem-solving stance directed at the same issues and is considered more adaptive. We also administered the Attentional Control Scale (ACS),^31^ a 20-item measure that probes the capacity to concentrate despite distracters or distress. Low ACS scores have been previously associated with increased depressive and anxiety symptoms, worry and difficulties redirecting attention away from anxious and ruminative thoughts.^31, 32, 33, 34, 35^ The RRS and ACS were administered immediately following the flanker task, and were added to the assessment battery shortly after initial data collection; thus, 23 out of 32 participants completed these measures. Finally, the Mini International Neuropsychiatric Interview (MINI)^36^ was used to assess comorbid diagnoses (see Beard and Björgvinsson^37^ for details on training and reliability).

### Flanker task

Participants completed an arrow flanker task.^38, 39^ On each trial, flanking arrows appeared first (duration: 100 ms) and were then joined by a center arrow (50 ms) for a total stimulus duration of 150 ms. On congruent trials, the center and flanking arrows pointed in the same direction (<<<<< or >>>>>), whereas on incongruent trials they pointed in opposite directions (<<><< or >><>>). The task was to indicate whether the central arrow pointed left or right by pressing one of two buttons on a keyboard as quickly as possible. Stimulus presentation was followed by a fixation cross (1400 ms). Each participant completed 30 practice trials (15 congruent and 15 incongruent) followed by five blocks of 70 trials (46 congruent and 24 incongruent). To ensure that enough errors were committed to allow analyses of post-error behavioral adjustments, block-by-block feedback was added after data collection had started (see Supplementary Material for details).

The main variables of interest were post-error accuracy (=% correct/total trials following an incorrect response) and reaction time (RT), as well as post-correct accuracy (=% correct/total trials following a correct response) and RT. Moreover, post-error adjustments were captured by computing the Laming effect (Accuracy_After Incorrect Trials_−Accuracy_After Correct Trials_) and the Rabbitt effect (RT_After Incorrect Trials_−RT_After Correct Trials_).^40, 41^ Positive Laming and Rabbitt effects index more adaptive post-error behavioral adjustments. As in prior studies, the post-error analyses were limited to trials that followed incongruent errors in order to disentangle post-error adjustments from congruency sequence effects.^10, 42^

### Data analysis

To evaluate whether treatment reduced symptoms, paired *t*-tests were performed on CESD-10 and ACS total scores. For the BASIS-24, a multivariate analysis of variance (ANOVA) was performed with Time (Time 1 and Time 2) and BASIS Subscore (depression functioning, relationships, self-harm, emotional lability, psychosis and substance abuse) as factors. Similar Time × CBT Subscore (behavioral activation and cognitive restructuring) and Time × RRS Subscore (brooding and reflective) multivariate ANOVA examined effects of treatment on CBT skill use and rumination. To determine whether treatment affected post-error behavioral adjustments, separate Time (Time 1 and Time 2) × Condition (post-error and post-correct) ANOVAs were run for accuracy and RT. Throughout the analyses, significant effects were followed up with paired *t*-tests.

Hierarchical regressions were run to test the hypothesis that baseline post-error adjustments (Laming or Rabbitt effect) would predict treatment outcome, as measured by the CESD-10, ACS and RRS. In the first set of regressions, Time 2 CESD-10 score was the criterion variable, and Time 1 CESD-10 score and T1 post-error effects were entered as predictors in the first and second steps, respectively. In the second set of regressions, Time 2 RRS subscore (brooding or reflection) served as the criterion variable. Time 1 RRS subscore, Time 1 CESD-10 score and Time 2 CESD-10 score were entered as predictors in the first, second and third steps, respectively, and the Time 1 post-error effects were entered in the fourth step. This approach investigates the relationship between the post-error effect (at Time 1) and rumination (at Time 2) while controlling for rumination at Time 1 and depressive symptoms at both times. The third set of regressions was identical, except that ACS scores were used in place of RRS scores. Significant effects were followed up with separate regressions for post-correct and post-error responses. In light of prior findings highlighting differences between depressed and control sample in post-error accuracy (rather than RT),^9, 10, 42^ we expected that the Laming effect and post-error (but not post-correct) accuracy scores would show significant findings. Moreover, we expected the (baseline) Laming effect to predict maladaptive (brooding) but not adaptive (reflection) forms of rumination at discharge.

### Results

See Supplementary Material for data on the basic flanker effects, indicating the task elicited the intended effects.

#### Treatment effects

Treatment efficacy: Table 1 summarizes clinical measures at admission and discharge (see Supplementary Material for details). In brief, CESD-10, BASIS subscales (depression, self-harm and psychosis), ACS, RRS brooding and CBT skills significantly improved from Time 1 to Time 2. These results support the efficacy of the treatment.

**Figure 1:**
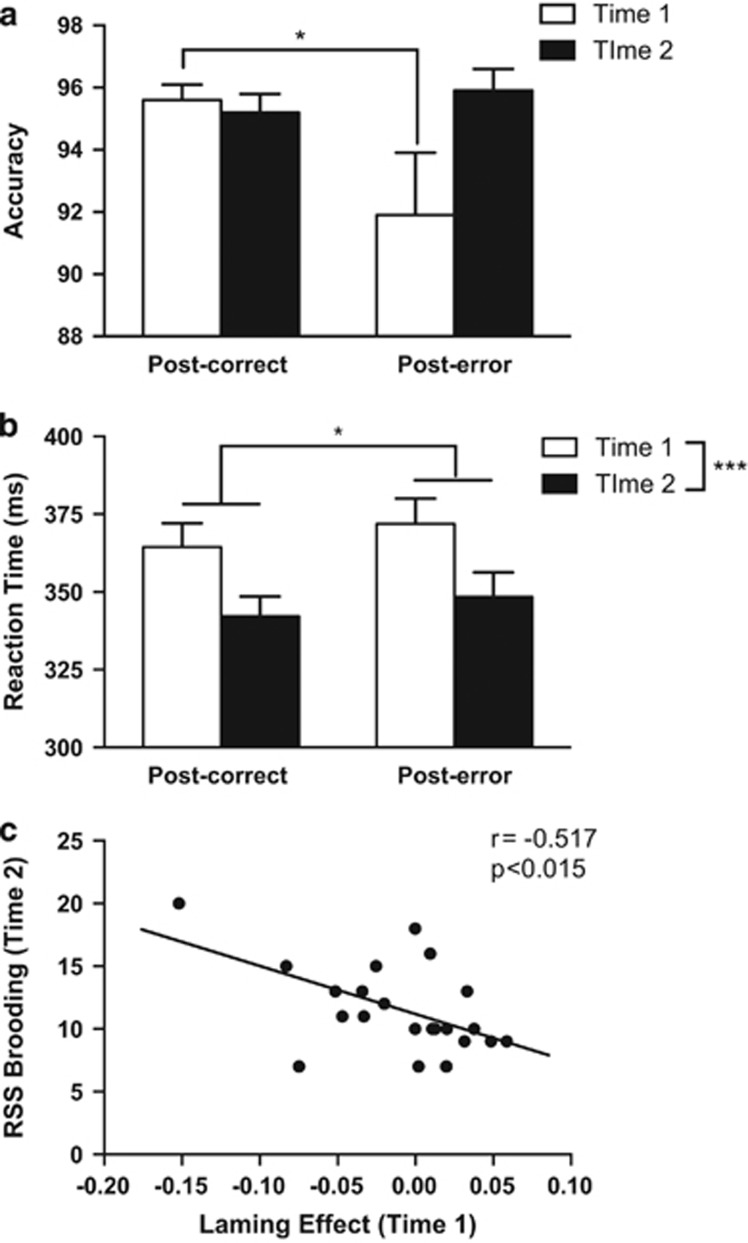
Post-correct and post-error (**a**) accuracy and (**b**) reaction time scores (in ms) at Time 1 (first day of admission to treatment program) and Time 2 (post treatment) for patients. *P<0.05, ^***^P<0.001, *post hoc t*-tests. (**c**) Correlation between Time 1 post-error accuracy and Time 2 RRS (Ruminative Responses Scale) brooding.

Post-error adjustments: accuracy: As shown in Figure 1a, post-error accuracy increased from Time 1 to Time 2, but there was little change in post-correct accuracy. The Time × Condition ANOVA returned a significant interaction (F(1,31)=4.75, *P*=0.037). The interaction remained significant when a between-subject factor was included to differentiate patients tested with (*n*=23) versus without (*n*=9) block-by-block feedback (F(1,30)=14.24, *P*=0.001). No other effects were significant. *Post hoc* *t*-tests showed that at Time 1, post-error accuracy (0.92±0.02) was significantly lower than post-correct accuracy (0.96±0.01) (*t*(31)=−2.10, *P*=0.044). By Time 2, this difference was not significant (*t*(31)=1.07, *P*>0.29). Accordingly, the Laming effect increased from Time 1 (−0.04±0.02) to Time 2 (0.01±0.01) (*t*(31)=2.18, *P*=0.037). Post-error accuracy was also higher at Time 2 (0.96±0.01) than Time 1 (0.92±0.02), although this effect was only marginally significant (*t*(31)=1.88, *P*=0.07).

Post-error adjustments: RT: As shown in Figure 1b, RT was faster at Time 2 (345.34±6.93) than Time 1 (368.14±7.66 ms), and for post-correct (353.33±6.59 ms) versus post-error (360.16±7.60) trials. Accordingly, the Time × Condition ANOVA revealed significant effects of Time (F(1,31)=27.09, *P*<0.001) and Condition (F(1,31)=5.73, *P*=0.023). The interaction was, however, not significant (F<1, *P*=0.78).

#### Regression analyses

None of the regression analyses testing the Rabbitt RT effect as a predictor were significant, and none of the analyses predicting Time 2 CESD-10 scores were significant. Thus, post-error behavioral adjustments measured at baseline did not predict treatment outcomes as measured by the CESD-10.

Post-error adjustments: accuracy: The Time 1 Laming effect was a negative predictor of RRS brooding scores (*β*=−0.520; Δ*R*^2^=0.24, ΔF(1,17)=5.88, *P*=0.027) and a positive predictor of ACS scores (*β*=0.370; Δ*R*^2^=0.11, ΔF(1,17)=4.53, *P*=0.048) at Time 2. The model considering adaptive forms of rumination (RRS reflection) as the criterion was not significant (*P*>0.54). Thus, larger Laming effects (that is, bigger post-error minus post-correct accuracy differences) at Time 1 predicted reduced brooding and increased attentional control at Time 2 after accounting for brooding and attentional control at Time 1 and depressive symptoms at both times.

To test the specificity of these findings, four additional hierarchical regressions were run in which the Laming effect was replaced by post-error or post-correct accuracy. RRS brooding was significantly predicted by post-error accuracy (*β*=-0.561; Δ*R*^2^=0.25, ΔF(1,17)=6.30, *P*=0.022) but not by post-correct accuracy (*β*=−0.167; Δ*R*^2^=0.021, ΔF(1,17)=0.41, *P*>0.53). Moreover, the Pearson correlation coefficient between Time 1 post-error accuracy and Time 2 RRS brooding was significant (*r*=−0.470, *P*<0.035; Figure 1c), but there was no such correlation with Time 1 post-correct accuracy (*r*=−0.08, *P*>0.60). Furthermore, these two dependent correlations were significantly different (*Z*=1.91, 1-tailed, *P*<0.05). Along similar lines, ACS scores were significantly predicted by post-error accuracy (*β*=0.407; Δ*R*^2^=0.12, ΔF(1,17)=5.13, *P*=0.037) but not by post-correct accuracy (*β*=0.111; Δ*R*^2^=0.01, ΔF(1,17)=0.36, *P*=0.55). This is notable because it suggests that improvements in post-error adjustment—rather than a global improvement in performance—are responsible for the observed relationships with attentional control and brooding.

## Study 2: Rodents

### Animals

The novel analysis described here was performed on a data set used previously to study attention as reflected by a variety of more traditional metrics, as described.^15^ In brief, a total of 11 male Sprague–Dawley rats (Charles River Laboratories, Raleigh, NC, USA) weighing 250–275 g were used for the analysis. Rats were housed in pairs and kept on a 12-h light–dark cycle with lights on at 0700 hours. All behavioral procedures were conducted during the light cycle. Rats were food restricted to 85% of their free-feeding weight throughout the experiment and had free access to water. The experiments were approved by the McLean Hospital Institutional Animal Care and Use Committee and conducted in accordance with National Institutes of Health guidelines.

### 5CSRTT

Rats were trained to detect the location of a brief stimulus light (0.5 s) presented randomly in one of five apertures, and nose-poke in the correct aperture within 5 s of the stimulus light presentation. Correct responses were rewarded with a food pellet (45 mg; BioServ, Frenchtown, NJ, USA) delivered to a food reward receptacle on the opposite wall. Incorrect responses and omissions resulted in a 5-s timeout. Tests consisted of 90 trials or 30 min, whichever came first. The criteria for stable responding was >60% correct responses and <20% omissions for five consecutive days.

### Surgical procedures

Once rats reached criteria, they underwent surgery to implant an intracerebroventricular cannula for drug delivery. Rats were anesthetized with pentobarbital (65 mg kg^−1^) and placed in a stereotaxic instrument. A stainless steel guide cannula (23 gauge, Plastics One, Roanoke, VA, USA) with a dummy stylet extending 1.5 mm beyond the cannula tip was lowered into the right lateral ventricle (relative to bregma: −0.8 mm anteroposterior, 1.3 mm mediolateral and −3.5 mm ventral to dura) and attached to the skull.

### Drugs and experimental design

After 5–7 days of recovery and restabilization of performance, rats first received a microinfusion of vehicle (artificial CSF; Harvard Apparatus, Holliston, MA, USA) to obtain baseline metrics. Following restabilization between infusions, rats (*n*=6) were then infused with CRF over a range of doses to identify those that produce minimal, intermediate and asymptotic responses (0.25–1.0 μg; American Peptide, Sunnyvale, CA, USA). All infusions were delivered over a 2-min period at a rate of 0.5 μl min^−1^. Rats were tested in the 5CSRTT 60 min after infusion. In a separate cohort, rats (*n*=5) received an intraperitoneal injection of JDTic (10 mg kg^−1^; Research Triangle Park, NC, USA) dissolved in 0.9% saline 48 h before CRF or vehicle (VEH) infusions to determine whether systemic administration of the KOR antagonist JDTic blocks the disruptive (depressive-like) effects of CRF.

### Data analysis

Error analysis was limited to data collected after treatment with vehicle or 0.5 μg CRF, because this dose was shown to induce intermediate performance deficits in traditional 5CSRTT metrics that were attenuated by JDTic pretreatment.^15^ Other doses tested previously produced negligible or asymptotic effects. To determine whether CRF treatment in rats causes deficits in post-error adjustments as described in depressed samples (see also Holmes and Pizzagalli^10^), post-error accuracy (=% correct/total trials following an incorrect response) and post-correct accuracy (=% correct/total trials following a correct response) were calculated. A Treatment (VEH and CRF) × Condition (post-error and post-correct) ANOVA was run separately for accuracy and RT with repeated measures on Treatment. Effects of CRF on post-error accuracy was analyzed with preplanned contrasts (Bonferroni tests) between VEH and CRF treatment days based on a specific *a priori* hypothesis that CRF treatment would produce similar depressive-like post-error adjustments as seen in humans. A similar analysis was run for the studies involving JDTic pretreatment.

### Results

**Figure 2:**
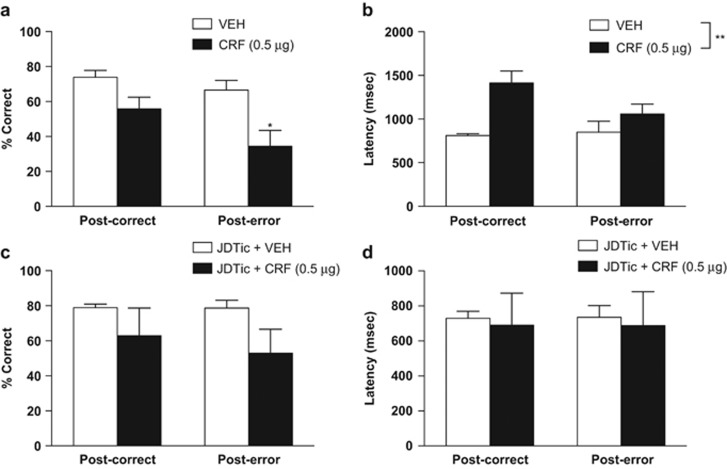
Post-correct and post-error (**a**) accuracy (reflected by % correct) and (**b**) latency (in ms) for vehicle (VEH)-treated and corticotropin-releasing factor (CRF)-treated rats, and (**c**) accuracy and (**d**) latency for VEH and CRF-treated rats with JDTic pretreatment. *P<0.05, *post hoc* Bonferroni tests; ^**^P<0.01, main effect of Treatment.

Consistent with our *a priori* hypothesis that the stress-peptide CRF would impair post-error adjustments in a manner similar to what observed in depressed human subjects, preplanned (Bonferroni) contrasts between CRF- and VEH-treated rats revealed that CRF treatment decreased post-error accuracy (34.34±9.11) compared with VEH treatment (66.51± 5.64; *P*<0.01) without producing a significant change in post-correct accuracy (55.79± 6.71 and 73.91± 3.91, respectively; Figure 2a). The ANOVA revealed a main effect of Treatment (F(1,10)=18.24, *P*<0.002) and a marginal effect of Condition (F(1,10)=3.94, *P*=0.075). Unlike the human data, however, the Treatment × Condition interaction was not significant (F(1,10)=1.42, *P*=0.26). Moreover, CRF treatment increased latency regardless of Condition: while the Treatment × Condition interaction for RT was not significant (F(1,10)=3.79, *P*=0.08), there was a main effect of Treatment (F(1,10)=16.29, *P*=0.0024).

There were no significant differences between rats treated with JDTic and VEH, and rats treated with JDTic and CRF in post-correct or post-error accuracy (Figure 2c). The main effect of Treatment was not significant (F(1,10)=3.35, *P*=0.105), and neither was the interaction (F(1,10)=0.18, *P*=0.68). There were also no significant differences on post-correct or post-error latency (Figure 2d). The main effect of Treatment was not significant (F(1,10)=0.08, *P*=0.78) nor was the interaction (F(1,10)=0.0008, *P*=0.98). These results are consistent with accumulating evidence that KOR antagonism has antidepressant-like effects in rodent models used to study depression.^43^

## Discussion

Depression has been linked to impairments in cognitive control, particularly deficits in adjusting behavior after errors or negative feedback.^7, 8, 9, 10, 11^ However, the clinical concomitants and neurobiological substrates of this impairment are largely unknown. We used a translational, cross-species approach to examine the robustness of this impairment in a real-world depressed sample and in rodents, as well as whether treatment can improve post-error processing in both species. First, in the depressed sample, accuracy for post-error trials was significantly lower than for post-correct trials at Time 1, thus replicating prior findings in subclinical^9, 42^ and clinical samples recruited from the community (for example, Holmes and Pizzagalli,^10^ and Steffens *et al.*^11^). Second, we report the novel finding that CRF administration in rats, previously found to elicit a depression-like phenotype, significantly reduces accuracy after incorrect but not correct responses, providing evidence that error-processing deficits observed in depressed humans can be mimicked in a rodent model that elicits depression-like phenotypes. Third, as expected, provision of treatment in humans (CBT and pharmacological) and rodents (pharmacological) was effective in improving post-error accuracy.

To the best of our knowledge, this is the first report that treatment may impact post-error behavioral adjustment. Although the 4% increase in post-error accuracy in patients from pre- to post treatment was small in magnitude (*d*=0.457), it is comparable to the between-group difference observed in prior studies comparing post-error accuracy in patients with major depressive disorder and healthy controls.^10^ Additional analyses in the patient sample further suggested that impaired post-error adjustments predicted functional outcome after treatment. Although post-error accuracy at baseline did not predict depressive symptoms following treatment, it did predict both reduced brooding and increased attentional control at post treatment. These findings are noteworthy because the demand for cognitive control is higher on post-error versus post-correct trials. Selective relationships between post-error accuracy at baseline and treatment effects on RRS brooding and ACS scores suggest an important role for baseline cognitive control in treatment outcomes.

Post-error accuracy was decreased in rats given a manipulation known to produce hallmark signs of depression and anxiety, resembling the pattern seen in the patient study. Specifically, CRF (which produces signs of depression in humans and depressive-like signs in rodents^15^) degraded post-error accuracy. Notably, the disruptive effect of CRF on post-error accuracy was not evident in rats pretreated with JDTic, a KOR antagonist with antidepressant- and anxiolytic-like effects in rodents.^24^ Although the general patterns of responding seen after CRF alone and those seen after JDTic plus CRF have similarities, KOR antagonist treatment reduced the magnitude of impairment in post-error accuracy (49% reduction versus 33% reduction). Thus, consistent with the human data, intervention can mitigate impairments in cognitive control. To the extent that the basic behavioral patterns seen in rats treated with CRF resemble those seen in depressed humans,^18, 19, 20, 21^ our data suggest that KOR antagonism may relieve certain cognitive deficits that accompany mood disorders.

The current findings should be considered in the context of the study design. First, the patient data stemmed from a naturalistic treatment setting. This provides an important test of post-error behavioral adjustment in depression, as the sample was characterized by severe psychopathology, high comorbidity, medication use and suicidal ideation. Moreover, in line with the Research Domain Criteria approach, we did not limit our sample to a particular mood disorder diagnosis. Although these decisions maximize the external validity of the study and allow a test of post-error processing in a real-world sample, they introduce several important caveats. First, the treatment received by the patients was uncontrolled and included various types of medication and CBT groups. Moreover, given the sample size, we did not have adequate power to control for the various combinations of treatments. Still, the fact that hypothesized findings emerged in such heterogeneous sample is noteworthy. Second, because we utilized a naturalistic treatment sample without a control group, we cannot rule out the possibility that pre-post changes in symptoms were due to regression to the mean or demand effects. Given this study design, we also cannot determine which aspects of treatment were involved in any post-error processing improvement. In addition, it is possible that improvements in post-error performance were due simply to practice effects. However, the specificity of the accuracy findings with respect to trial type (post-error versus post-correct trials) and constructs (for example, maladaptive versus adaptive forms of rumination) makes demand or practice effects less likely. Nonetheless, future studies with a control psychiatric sample not receiving treatment are warranted. Third, the current real-world patient sample resulted in a greater percentage of patients not meeting data quality cutoffs (e.g., making enough, but not too many errors to examine these effects) compared with previous studies of unmedicated and comorbidity-free community samples. Thus, while our ‘real-world' sample enhances generalizability to comorbid, chronic and medicated patients, the higher exclusion rate may also limit generalizability. Importantly, patients excluded for quality cutoffs did not differ from included patients on baseline levels of depression, anxiety, functioning, CBT skills or demographic variables. Fourth, although depression was the focus of treatment, a number of the patients had comorbid anxiety disorders. Impairments in cognitive control, and specifically abnormal error processing, have also been implicated in anxiety disorders (for example, Weinberg *et al.* ^44^). Thus, it is possible that anxiety symptoms also contributed to the abnormal error processing observed in the current study.

Finally, the patient study and rodent study were not designed as complementary experiments. Although we acknowledge that these rodent studies were not specifically intended to examine post-error performance, reanalysis of our existing data demonstrates an important proof of concept without requiring the use of additional experimental animals, and highlights the translational power of post-error processing in models of depressive phenotypes across species. Because these data were from a previously published report in which we studied the mechanisms by which stress degrades attention,^15^ we had two separate cohorts of rats available for the reanalysis, which enabled us to examine the effects of a KOR antagonist on post-error performance. It is conceivable that more thorough dose–effect functions for both CRF and JDTic in tests specifically designed to examine post-error accuracy could reveal more prominent effects.

These limitations notwithstanding the current findings are novel and suggest several areas of future research. For example, as studies have shown that computer-based cognitive training may be able to improve cognitive functioning in patients with major depressive disorder,^45, 46^ such training may be especially beneficial for individuals with poor post-error behavioral adjustments. More generally, identification of cognitive domains in depressed humans that have analogs in rodents in depressive-like states has become a high priority for modeling mood disorders in laboratory animals.^47^ The neural substrates of these effects can be examined in great detail in rodents, with the goal of identifying brain structures or mechanisms that could be targeted by new types of therapeutic intervention. In this context, and in light of prior electrophysiological data linking impaired post-error processing with dysfunction within frontocingulate pathways,^10^ it will be important to test in future studies whether KOR antagonists might exert antidepressant effects by normalizing brain circuitry critically implicated in cognitive control.

## Figures and Tables

**Table 1:** Self-report measures in Study 1

	*Time 1*		*Time 2*		N	T-*value*	P-*value*
	*Mean*	*s.d.*	*Mean*	*s.d.*			
*RRS*							
Brooding Subscore	14.26	2.88	11.52	3.44	23	3.497	0.002
Reflection Subscore	12.87	2.55	12.39	2.50	23	0.794	0.436
Total	63.57	8.61	53.17	11.10	23	3.707	0.001
ACS	43.57	9.58	48.43	10.22	23	−3.063	0.006
CESD-10	18.65	6.15	9.81	5.69	31	8.398	0.001
							
*BASIS-24*							
Depression	2.58	0.83	1.59	0.72	31	8.231	0.001
Relationships	1.26	0.71	1.26	0.85	31	-.029	0.977
Self-harm	0.73	0.74	0.45	0.66	30	2.168	0.039
Emotional lability	1.58	1.05	1.36	0.91	30	1.383	0.177
Psychosis	0.40	0.80	0.20	0.49	29	2.159	0.040
Substance abuse	0.50	0.86	0.28	0.39	31	1.826	0.078
Total	1.73	0.61	1.13	0.39	28	6.647	0.001
							
*CBT Scale*							
Behavioral activation	17.10	5.18	24.90	5.94	30	−6.612	0.001
Cognitive restructuring	23.10	6.76	33.80	6.96	30	−7.631	0.001
Total	40.20	10.58	58.70	12.27	30	−7.563	0.001

Abbreviations: ACS, Attentional Control Scale; BASIS-24, Behavior and Symptom Identification Scale; CBT Scale, Cognitive-Behavioral Therapy Skills Questionnaire; CESD-10, Center for Epidemiologic Studies Depression Scale-10; RRS, Ruminative Responses Scale.

## Notes

WAC discloses that he is an inventor on a patent that claims the use of kappa-opioid receptor antagonists for the treatment of depression (assignee: McLean Hospital), and over the past 2 years he has received compensation for editorial duties from the American College of Neuropsychopharmacology. FIC is an inventor of US patents claiming the composition of JDTic owned by the Research Triangle Institute. Over the past 2 years, DAP has received consulting fees from Otsuka America Pharmaceutical, Pfizer and Servier for activities unrelated to the current research. All the remaining authors declare no conflict of interest.
