# Supplementary material for: Abnormal error processing in depressive states: a translational examination in humans and rats
Source: Transl Psychiatry. 2015 May 12;5(5):e564–. doi: 10.1038/tp.2015.54 (PMC4471285; doi:10.1038/tp.2015.54)

**Supplemental Information**

Abnormal Error Processing in Depressed States: A Translational Examination in Humans and Rats

Courtney Beard, Rachel J. Donahue, Daniel G. Dillon, Ashlee Van’t Veer, Chelsea Webber, Josephine Lee, Elyssa Barrick, Kean J. Hsu, Dan Foti, F. Ivy Carroll, William A. Carlezon Jr.,

**Thröstur Björgvinsson,** & Diego A. Pizzagalli

**Supplemental Methods**

*Additional methodological details for the flanker arrow task.* To ensure adequate task difficulty, a response threshold was instituted. Specifically, if the RT on a trial was slower than the 85^th^ percentile of the incongruent RT distribution from the preceding block, a screen reading “TOO SLOW!” was presented for 300 ms immediately following the fixation cross. If the RT was below this threshold, the “TOO SLOW!” screen was omitted and the fixation cross remained onscreen for the 300 ms interval (see Supplement Figure S1). Finally, all trials ended with an additional 200-400 ms of fixation, yielding a total trial duration of 2050-2250 ms. The sequence of congruent and incongruent trials was established using optseq2 (<http://surfer.nmr.mgh.harvard.edu/optseq/>) and was the same for all participants. To ensure that enough errors were committed to allow analyses of post-error behavioral adjustments, block-by-block feedback was added after data collection had started. Specifically, if a participant committed less than three incongruent errors in a given block, the instructions “*Remember to respond as QUICKLY as possible while still being accurate*” were presented after the block. Conversely, the instructions “*Remember to respond as ACCURATELY as possible while still being fast*” were presented if a participant committed six or more incongruent errors in a given block. If none of these conditions occurred, the instruction “*Please respond as quickly and accurately as possible*” were provided after the block.

Quality control measures (established *a priori*) were used to exclude datasets characterized by poor performance. We first identified trials with RT outliers, defined as raw RTs shorter than 150 ms or log-transformed RTs that exceeded the participant’s mean±3SDs (computed separately for congruent and incongruent trials). We then excluded datasets with more than 35 RT outliers (i.e., 10% of trials), fewer than 200 congruent or 90 incongruent outlier-free trials, or fewer than 50% correct responses for either congruent or incongruent trials. Because of our focus on post-error adjustments, we also excluded datasets with fewer than 6 errors on incongruent trials, which may be the lower limit for studying error-related processes in brain and behavior (e.g., Olvet & Hajcak, 2009). Trials characterized by RT outliers were excluded from all analyses for datasets that passed these quality control checks.

**Supplemental Results**

*Task manipulation check*. To evaluate whether the Flanker task elicited the intended effects, paired t-tests were run to compare accuracy and RT scores for congruent and incongruent trials at Time 1. Relative to congruent trials, incongruent trials were associated with significantly lower accuracy and significantly slower RT (both |ts| > 11.08, both ps < 10^-12^), indicating that the task elicited the expected Flanker interference effects.

*Detailed results for treatment efficacy.* CES-D-10 scores were significantly lower at Time 2 versus Time 1. With respect to the BASIS-24**©**, the MANOVA revealed significant main effects of *Time* and *Subscore*, as well as a significant interaction (all Wilks' Lambda > 10.77, all ps < 0.001). Post-hoc tests revealed significant improvements for the Depression Functioning, Self Harm, and Psychosis subscores, with trend-level improvement for Substance Abuse. There was no change over time in Relationship Quality, and a numerical decline in Emotional Lability did not approach significance. For the CBT-Skills Questionnaire, the main effects of *Time* and *Subscore* and their interaction were significant (all Wilks' Lambda > 10.04, all ps < 0.005). Both subscores were significantly higher at Time 2 than Time 1, suggesting that participants acquired skills imparted in their CBT sessions. Finally, for the RSS, the MANOVA revealed significant main effects of *Time* and significant interactions (Wilks' Lambda > 7.55, ps < .012). Time 1 RRS brooding was significantly higher than RRS Reflection, t(22)=2.16, p <.045, whereas these variables did not differ at Time 2 (p > 0.20). Moreover, RRS Brooding showed a significant reduction from Time 1 to Time 2, whereas RRS Reflection did not differ significantly between the two sessions. Along similar lines, ACS scores increased from Time 1 to Time 2.

*Relationships among task performance and clinical data*. Upon request by an anonymous reviewer, interrelations between task performance (post-error accuracy/RT, post-correct accuracy/RT, laming effect, and rabbit effect) and clinical data (CESD-10, BASIS, CBT Scale) were computed for Time 1 (Table S1), Time 2 (Table S2), and Change from T1-T2 (Table S3).

**Table S1.** Correlations among Self-report and Flanker variables at T1 (patient sample)

|  | 1 | 2 | 3 | 4 | 5 | 6 | 7 | 8 | 9 | 10 | 11 | 12 | |
| --- | --- | --- | --- | --- | --- | --- | --- | --- | --- | --- | --- | --- | --- |
| 1. post-error accuracy |  |  |  |  |  |  |  |  |  |  |  | |  |
| 2. post-error RT | -.222 |  |  |  |  |  |  |  |  |  |  | |  |
| 3. post-correct accuracy | .503** | .117 |  |  |  |  |  |  |  |  |  | |  |
| 4. post-correct RT | -.34 | .879** | .079 |  |  |  |  |  |  |  |  | |  |
| 5. Laming effect | .962** | -.286 | .249 | -.405* |  |  |  |  |  |  |  | |  |
| 6. Rabbitt effect | .193 | .383* | .09 | -.104 | .188 |  |  |  |  |  |  | |  |
| 7. ACS | -.18 | -.27 | -.023 | -.219 | -.201 | -.212 |  |  |  |  |  | |  |
| 8. RSS Brooding | -.12 | .615** | .12 | .523* | -.22 | .441* | -.126 |  |  |  |  | |  |
| 9. RSS Reflective | -.36 | .316 | -.06 | .261 | -.399 | .241 | .141 | .358 |  |  |  | |  |
| 10. CESD-10 | .204 | .192 | .046 | .044 | .214 | .315 | -.478* | .442* | .02 |  |  | |  |
| 11. BASIS-24 | .303 | .196 | .106 | .032 | .305 | .34 | -.335 | .538* | -.012 | .828* |  | |  |
| 12. CBT-Behavioral Activation | -.34 | -.043 | -.434* | .024 | -.245 | -.135 | .336 | .272 | .355 | -.194 | -.162 | |  |
| 13. CBT-Cognitive Restructuring | -.479** | -.051 | -.453** | .136 | -.394* | -.368* | -.014 | -.123 | .289 | -.082 | -.187 | | .551* |

Note. * p < .05, **p <.01

**Table S2**. Correlations among Self-report and Flanker variables at T2 (patient sample)

|  | 1 | 2 | 3 | 4 | 5 | 6 | 7 | 8 | 9 | 10 | 11 | 12 |
| --- | --- | --- | --- | --- | --- | --- | --- | --- | --- | --- | --- | --- |
| 1. post-error accuracy |  |  |  |  |  |  |  |  |  |  |  |  |
| 2. post-error RT | .565** |  |  |  |  |  |  |  |  |  |  |  |
| 3. post-correct accuracy | .526** | .514** |  |  |  |  |  |  |  |  |  |  |
| 4. post-correct RT | .445* | .925** | .492** |  |  |  |  |  |  |  |  |  |
| 5. Laming effect | .653** | .176 | -.301 | .061 |  |  |  |  |  |  |  |  |
| 6. Rabbitt effect | .514** | .626** | .287 | .284 | .320 |  |  |  |  |  |  |  |
| 7. ACS | -.014 | -.116 | .165 | -.111 | -.170 | -.063 |  |  |  |  |  |  |
| 8. RSS Brooding | -.249 | .090 | .353 | .150 | -.633** | -.099 | -.033 |  |  |  |  |  |
| 9. RSS Reflective | -.212 | -.006 | -.086 | .018 | -.180 | -.056 | -.117 | .388 |  |  |  |  |
| 10. CESD-10 | -.206 | -.054 | -.053 | .057 | -.181 | -.246 | -.297 | -.095 | .084 |  |  |  |
| 11. BASIS-24 | -.440* | -.035 | -.124 | .073 | -.377* | -.234 | -.399 | .221 | .379 | .668** |  |  |
| 12. CBT-Behavioral Activation | .336 | .264 | .342 | .097 | .071 | .456** | .318 | .030 | -.213 | -.707** | -.588** |  |
| 13. CBT-Cognitive Restructuring | .275 | .198 | .131 | .021 | .188 | .446* | .066 | -.194 | -.277 | -.724** | -.461* | .807** |

Note. * p < .05, **p <.01

**Table S3**. Correlations among Change Scores for Self-report and Flanker variables (patient sample)

|  | 1 | 2 | 3 | 4 | 5 | 6 | 7 | 8 | 9 | 10 | 11 | 12 |
| --- | --- | --- | --- | --- | --- | --- | --- | --- | --- | --- | --- | --- |
| 1. post-error accuracy |  |  |  |  |  |  |  |  |  |  |  |  |
| 2. post-error RT | -.193 |  |  |  |  |  |  |  |  |  |  |  |
| 3. post-correct accuracy | .335 | -.062 |  |  |  |  |  |  |  |  |  |  |
| 4. post-correct RT | -.492** | .632** | -.026 |  |  |  |  |  |  |  |  |  |
| 5. Laming effect | -.949** | .185 | -.02 | .513** |  |  |  |  |  |  |  |  |
| 6. Rabbitt effect | .326 | .480** | -.044 | -.376* | -.360* |  |  |  |  |  |  |  |
| 7. ACS | .155 | .086 | -.003 | .504* | -.189 | -.409 |  |  |  |  |  |  |
| 8. RSS Brooding | .041 | .168 | .134 | .37 | .049 | -.162 | .492* |  |  |  |  |  |
| 9. RSS Reflective | -.556** | .195 | -.505* | -.037 | .3 | .298 | .261 | .418- |  |  |  |  |
| 10. CESD-10 | -.143 | .367* | -.338 | .2 | .038 | .178 | .28 | .31 | .408 |  |  |  |
| 11. BASIS-24 | .053 | .357 | -.163 | .199 | -.108 | .159 | .477* | .479* | .431 | .802* |  |  |
| 12. CBT-Behavioral | -.008 | -.034 | -.039 | .033 | -.005 | -.078 | .386 | .468* | .473* | .305 | .339 |  |
| 13. CBT-Cognitive Res. | .199 | .051 | -.017 | -.092 | -.207 | .166 | .233 | .408 | .349 | .209 | .175 | .763** |

Note. * p < .05, **p <.01

**Figure S1.** Arrow Flanker Task used in Study 1 (patient sample)


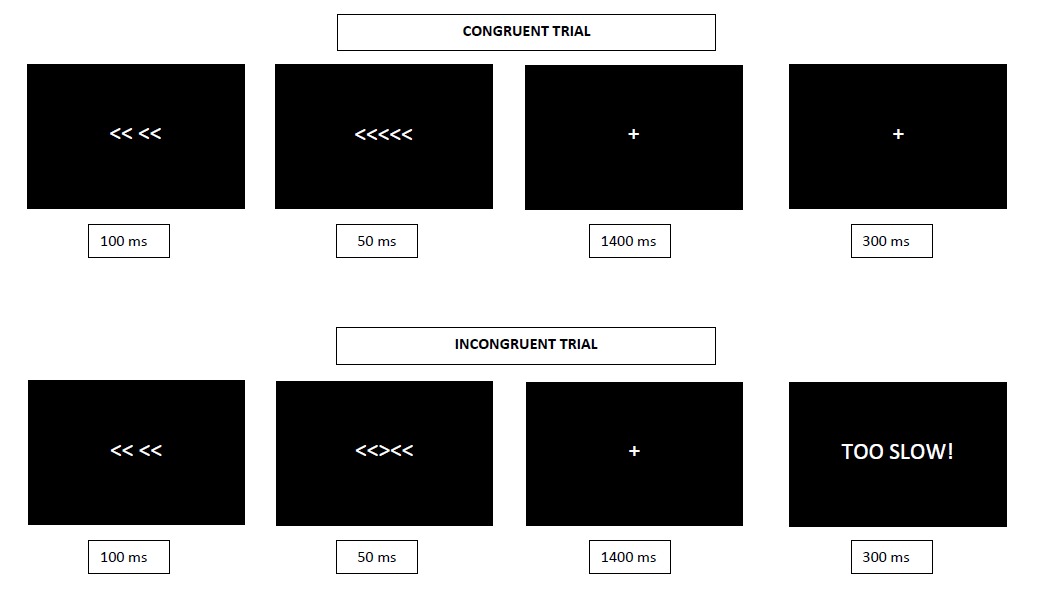

Supplement: Supplementary Information [file tp201554x1.doc]
